# Efficiency comparison of natural coagulants (Cactus pads and *Moringa* seeds) for treating textile wastewater (in the case of Kombolcha textile industry)

**DOI:** 10.1016/j.heliyon.2025.e42379

**Published:** 2025-02-10

**Authors:** Getahun Demeke Worku, Shimeles Nigussie Abate

**Affiliations:** Department of Chemical Engineering, Kombolcha Institute of Technology, Wollo University, Ethiopia

**Keywords:** Textile wastewater, Coagulation, Coagulant, Wastewater treatment for cactus pads and *Moringa* seeds

## Abstract

The issue of water contamination has gotten worse worldwide due to industrialization and population increase. The wastewater discharge techniques used by Ethiopia's textile sector do not comply with the national discharge standard. Because the discharge of inadequately treated or untreated textile effluent into the environment is becoming more frequent. Thus, minimizing this trend's harm to the environment requires using the best wastewater treatment technology. Coagulation is considered one of the most effective methods for treating textile wastewaters, which technically need to have a natural or artificial coagulant added to eliminate dangerous pollutants. Nonetheless, criticism has been leveled against the use of chemical coagulants due to their serious disadvantages. This has led to an interest in substituting natural materials for chemical coagulants. The main objective of this study was to evaluate and compare the effectiveness of various powders and *Moringa* seed cactus pad powders when employed as a natural coagulant to clean up textile effluent. *Moringa* seeds and cactus pad consist of proteins (cationic and dimeric) which help in neutralizing and absorbing colloidal charges in water containing suspended solids. To examine the impacts of various operating parameters, including coagulant dose, pH, and mixing time, as well as the ideal coagulation conditions, experiments were conducted using a jar testing system. The ideal parameters were pH 4, 5 g/l of coagulant, 40 min of mixing time, 2 min of fast mixing at 100 rpm, 40 min of gradual mixing at 40 rpm, and 30 min of settling time. Under these ideal circumstances, 82.33 Moreover, cactus pad powder reduced turbidity by 53.16 %, TDS by 97 %, BOD by 58.75 %, COD by 25.37 %, Conductivity by 60 %, and TSS by 56.33 %. Therefore, using *Cactus pad* as a natural coagulant is advised. Rather than using *Moringa seed*, it efficiently eliminates turbidity, TDS, and TSS from wastewater effluents from the textile sector. Eventually, one of the natural coagulants employed in the coagulation process, *Cactus pad*, proved to be useful and was chosen.

## Graphical abstract

**Figure:**
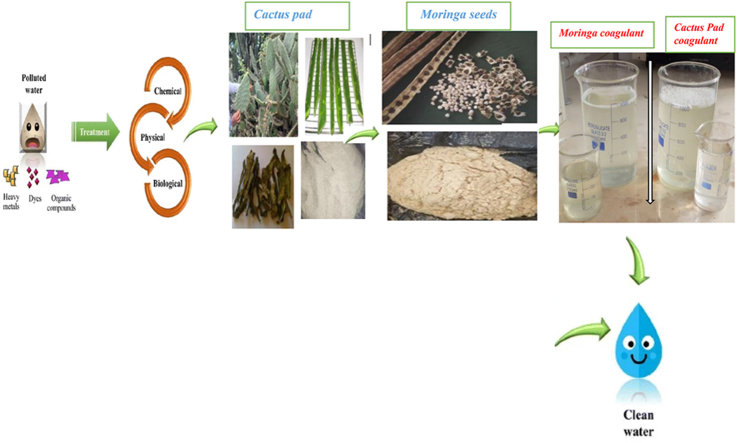

## 1 Introduction

One of the biggest issues facing the entire world today is water contamination brought on by the textile industries' failure to dispose of their wastewater properly. In several nations, like China and the estuaries of South Africa, the textile industry significantly contributes to both environmental pollution and the global economy [1]. Economic expansion stems from people's desire for better lives, which in turn causes environmental contamination [2]. The textile industry is one of the most important and widespread industries in Ethiopia.

For centuries, humanities have relied on traditional chemical coagulants, such as ferric chloride and alum, to treat contaminated water. They've got a great track record of handling other things, clarifying things, and clearing out all those floaty pieces. They agitate these little particles until they group to form larger chunks. These fragments are then removed by filtering out or sinking to the bottom. The problem is that employing chemicals isn't always easy. It frequently entails costly maintenance, difficulties disposing of sludge waste, and concerns about any residual toxins that could harm the environment or human health.

As is well known, Moringa seeds and cactus fragments are becoming increasingly popular as eco-friendly alternatives due to their affordability and ease of usage. Proteins and polysaccharides, which are found in these natural aids, appear to have the ability to effectively target and eliminate corrupt material. According to recent studies, they can handle filth from live organisms and harsh wastewater from textiles that are dye-loaded [3].

According to Ref. [4] there is an abundance of dangerous contaminants in textile effluent, both in terms of volume and substance. This is troubling because it is estimated that the industry will require 200 L of water to create 1 kg of textiles. However, textile effluents that contain non-biodegradable components, are salinized, or have brilliant colors may have a high biological and chemical oxygen demand [5]. It also includes trace levels of metals like copper, zinc, arsenic, and chrome along with oils, greases, and waxes [6]. One efficient method for treating textile wastewater is coagulation [7]. This method has been verified to eliminate organic materials that raise the BOD and COD contents of wastewater, as well as dissolved, suspended, and colored dyes that cause turbidity [8]. To solve the issues with chemical coagulants, more natural coagulants must be used in the treatment of drinking water. Generally speaking, naturally occurring coagulants are thought to be harmless for human health. Numerous natural coagulants were created or isolated from microbes, animals, or plants as a result of some research that was done on the subject. *Moringa* seeds are among the substitutes. An excellent illustration of a so-called "multipurpose tree" is the *Moringa.* According to earlier research, *Moringa* is non-toxic and should be used as a coagulant in impoverished nations [9].

Many benzyl isothiocyanate and benzyl glycosylate compounds, which have antibacterial properties, are present in the seed. The seed is thought to be an organic, naturally occurring polymer. Dimer proteins are the active components. The protein powder is completely soluble in water and stable. Several theories have been proposed to explain the coagulation mechanism of *Moringa* oleifera coagulant protein [10]. It has been observed that the antibacterial capabilities of *Moringa* seeds allow a recombinant protein to flocculate both Gram-positive and Gram-negative bacterial cells. Here, microbes can be eliminated by settling in the same way as colloids in appropriately coagulated and flocculated water are eliminated. However, the seeds may potentially directly affect microbes, which would impede their growth. Antimicrobial peptides are believed to function by causing cellular disruption [11].

Although there are several ways to remediate textile wastewater, most of them are either very expensive or hazardous to the environment. Since ancient times, a variety of chemical coagulants, including aluminum salt, ferric salt, and synthetic polymers, have been used extensively to cleanse and purify water. These chemical coagulants discharge toxic compounds into the environment that are detrimental to human health, according to Ref. [12]. Additionally, alum has caused some problems, including (1) its reaction with the natural alkalinity of the water, which lowers the pH (lime is added to correct the pH, which is thought to be an extra expense for water treatment firms); and (2) its poor coagulation performance in cold waters. Furthermore, the optimal application of alum requires technical know-how and training. Finding more alternative coagulants is crucial to reducing these problems. At the moment, the focus is on locating renewable resources [13]. In order to create a novel coagulant, the usefulness of *Moringa* seeds and cactus leaves is compared in this study. *Moringa* seed and cactus mucilage that can be found nearby could be utilized as coagulants. Therefore, one of the greatest and most effective methods for treating textile wastewater is coagulation using a natural coagulant. To achieve sustainable development, turbidity, TDS, and TSS removal from textile effluent is crucial. This study examines the efficacy of *Moringa* seed and cactus pad as natural coagulants. These substances are well renowned for being affordable and eco-friendly. Turbidity, TDS, and TSS are also successfully eliminated by them. The scope of the study focused on the Kombolcha Textile Industry. It assesses the coagulation efficiency of cactus pads and Moringa seeds in coagulating textile wastewater under various conditions. It looks at both natural coagulants and the dosages and operating conditions that would ensure maximum efficiency.

This study could have several significant outcomes. Promoting the use of local and sustainable coagulants is one. With this study, we also hope to promote the adoption of natural coagulants in the treatment of industrial effluents. Still, the most important outcome of this study is the potential provision of information on eco-friendly coagulants and the integration of these coagulants into the treatment of industrial wastewater, which could reach the scale of 'emerging technology.

## 2 Materials and methods

### 2.1 Raw materials and equipment

The extraction of a natural coagulant from *Moringa* seeds for the treatment of textile wastewater is described in this section. Experiments were carried out in the Department of Chemical Engineering laboratory at Kombolcha Institute of Technology (Kiot) to create a natural coagulant from *Moringa* seed for the purpose of treating textile wastewater.

The laboratory equipment used includes a Drying Oven, which removes moisture from goods, and Gloves to ensure hand safety during operations. A Volumetric Cylinder is employed to measure and tally the volumes of products and samples, while Plastic Sacks and Beakers are utilized for collecting and storing samples from various sources. For consistent mixing, a Stirrer with a Magnetic Bar is used. The Weight Balance accurately measures sample weights, and a Desiccator cools down dried samples to prevent moisture reabsorption. To reduce raw material particle sizes, a Domestic Mill is applied, followed by a Screener or Sifter for analyzing and separating materials based on particle sizes. Filter Paper aids in separating solids from liquids during sample preparation. The pH Meter measures the acidity or basicity (pH) of samples, and a HACH Turbidity Gauge is used to test and measure the turbidity of water samples.

### 2.2 Chemicals and reagents

Many chemicals were used in the experiment, one of which being distilled water, which was used to clean the raw materials and make reagents for turbidity testing. To maintain good hygiene, detergent was used to clean hands. The main raw materials used in the studies were cactus pads and *Moringa* seeds. In addition, the pH of the sample water was adjusted using a buffer solution with a pH of 4.7. Oil was extracted from the powdered *Moringa* seeds using hexane. The chemical engineering department at Wollo University has easy access to all of these compounds in its chemistry laboratory.

### 2.3 Raw material and sample collection

The primary raw materials for this study were mature, healthy *Moringa* seeds and fresh cactus leaves. With a focus on a section of Ancharo near the Wollo University Kombolcha campus, the items were physically gathered from Kombolcha town. Additionally, a wastewater sample was collected from Kombolcha town's neighboring textile plant. An Alternative to Inorganic Chemicals, an industry wastewater treatment facility can process 3500 m^3^ of wastewater per day. Wastewater samples were taken from the treatment plant's inlet using grab sampling. To collect samples, clear polyethylene terephthalate (PET) containers were first washed with detergent, then rinsed with tap water, and then again with deionized water. After the samples were collected, they were kept at 4 °C at the Wollo University KIOT chemical engineering and BGI laboratory class to lessen the chance that their characteristics would change before the analysis was finished.

### 2.4 Characterization of textile wastewater

Grab sampling was used to collect wastewater samples from the treatment plant's inlet. Clean polyethylene terephthalate (PET) containers were cleansed with tap water, deionized water, and detergent before being used to gather samples. To reduce the possibility that the samples' features would change before the analysis was completed, they were stored at 4 °C in the Wollo University KIOT chemical engineering and BGI laboratory class after they were collected.

### 2.5 Methods for inlet and outlet wastewater quality tests

#### 2.5.1 Measurement of Turbidity

Procedure for Testing Turbidity: After adding a sample of water to the beaker, the turbidity meter was calibrated, placed into the sample, and the result was noted. Nephlometer Model 2100N/AN, which is readily available and makes measuring sample turbidity easy, was the approach employed for this study.

#### 2.5.2 PH Measurements

With the use of a pH meter, the sample's pH was measured. Before beginning the analysis, the pH meter was calibrated using buffer solutions with pH values of 4.0 and 7.0.

#### 2.5.3 Total solid analysis

The first part of the experiment was to put a clean dish in an oven set at a temperature between 103 °C and 105 °C for 1 h. This was to ensure that the dish that had just been washed with distilled water and soap was truly dry. Then the dish was taken out of the oven and put into a desiccator at room temperature. Put the dish in the oven for 1 h to evaporate the contents. The dish was quickly weighted after being cooled in desiccators to achieve temperature equilibrium. Equation (3.1) can be used to calculate the total solid quantities in the sample.

The formula for total solids mg is

3.1 $$\text{Total}\;\text{solids}\;\left({\text{mgL}}^{‐1}\right),=\frac{\left(A‐B\right)}{\text{Sample}\;\text{volume}\;\text{ml},}*\;1000$$

A is the weight of the dish plus the dried residue, and B is the dish's weight.

#### 2.5.4 Total dissolved solid analysis

Protocol for the Total Dissolved Solid Analysis Experimental.

1. Measured out a piece of the mixed sample and filtered it through filter paper. Gather the filtrate in a weight-evaporating plate that you had previously prepared.
2. Evaporated the contents by placing the dish in an oven for 1h.
3. Cooled the dish in desiccators to balance temperature and weighed it immediately.
4. The following formula can be used to calculate the total dissolved solid quantities in the sample:

3.2 $$\text{Total}\;\text{dissolved}\;\text{solid}\;\left({\text{mgL}}^{‐1}\right),=\frac{\left(A‐B\right)}{\text{Sample}\;\text{volume}\;\text{ml},}*\;1000$$

Where; A = weight of dried residue that passed the filter paper + dish, and B weight of the dish.

#### 2.5.5 Total suspended solid analysis

Total suspended solids consist of silt, clay, and small particles of inorganic and organic materials. The total suspended solids were determined using the following formula.

3.3 $$\text{Total}\;\text{Suspended}\;\text{Solid}\;\left({\text{mgL}}^{‐1}\right),=\text{TS}\;‐\;\text{TDS}$$

### 2.6 Methods coagulation experiments

The most popular experimental technique for assessing and improving coagulation-flocculation processes based on accepted techniques is the jar test [14]. Similarly, the batch test apparatus used in this study included three spindle steel paddles and three beakers. The wastewater samples were well mixed before the jar test, and the pH at baseline was adjusted using buffers 4 and 7. The beakers were filled with the powdered coagulants (*Moringa* seed and cactus pads) at different rates (2–8 g/l). The beakers were then mixed at different speeds and pH levels, alternating between slow mixing at 40 rpm for 20–60 min and rapid mixing at 100 rpm for 2 min. Rapid mixing disperses the coagulant uniformly throughout the beakers, whereas slow mixing promotes floc formation by increasing particle collisions, which leads to larger flocs [15].

The turbidity, TDS, and TSS of each beaker's supernatant sample were then measured. This test was used to determine the end concentration, which was then compared to the findings of a wastewater sample that had a starting concentration. The experiment's coagulant dose (2–8 g/l) and mixing time (20–60 min) were changed to investigate the effects of different operating factors on the coagulation-flocculation process and help achieve the ideal combination of parameters reproducible. Finding the optimal operating parameters that rely on increased turbidity TDS and TSS removal efficiency was the aim of this investigation. To ensure high reproducibility, each coagulation experiment was carried out in triplicate.

### 2.7 Methods coagulant preparation

The thorns and outer covering of the cacti were removed, and the samples were put in fresh, clean polyethylene plastic bags after being thoroughly cleaned in distilled water. The interior of the plant, or cactus mucilage, was gathered and dried in an oven set to a temperature of 65 °C. The composition of the mucilage may become denatured if the temperature rises over this point. After that, the dry material was stored for a whole day. A coffee mill was then used to grind the dried cactus material into a powder, and any particles smaller than 400 μm were removed by sieving. After being used for coagulation, this powder was stored at room temperature until the analysis. For this investigation, *Moringa* seed was manufactured concurrently. Considering that the *Moringa* seed can be used as a coagulant in two different ways: by using it as a regular seed or by extracting its oil (defatted cake). Some research have suggested that using defatted *Moringa* seed is important to improve the effectiveness of wastewater treatment [16]. According to this advice, defatted *Moringa* seed was made as follows: The seeds with pods on them were first manually separated. Since green seeds have little coagulation activity, the seeds with outer shells are dried in the sun. After being dried, seeds are then manually removed from their outer shells [17]. Following the drying of the seed kernel, the material was ground into a fine powder, about 400 μm in size, using a domestic mill to achieve the solubilization of the seed's active components [18]. The powder was then allowed to soak in hexane for 30 min at room temperature, stirring now and then. 500 ml of hexane was mixed with 100 g of the powder. To obtain the defatted cake, the solution must be filtered via filter paper [19]. The residual solids (process cake) are dissolved in water, stirred, and filtered before being dried for 24 h at 40 °C in an oven. This is useful in getting rid of any hexane that might be present in the powdered seed cake. Ultimately, the powdered dry seed was kept at room temperature until it was needed for coagulation. Generally, the flow diagrams of *Moringa* seed powder in defatted cake form preparation.

#### 2.7.1 Collection of Textile wastewater samples

The Kombolcha textile industries provided the study's sample waters. The wastewater was found to be more turbid and had a brownish-yellowish hue. Other raw water, on the other hand, had a reddish-yellow tint and was less turbid. As a result, wastewaters were gathered using plastic containers before starting experiments right away (see Table 1).

**Table 1:** Summarizing the experimental parameters.

Parameter	Value Range
Coagulant dose	2–8 g/L
pH Levels	4 and 7
Mixing Speed (Rapid)	100 rpm
Mixing Speed (Slow)	40 rpm
Mixing time (Rapid)	2 mini
Mixing Time (Slow)	20-60 mini

### 2.8 Experimental design and statistical analysis

Stat-Ease, Inc.'s Design-Expert statistical software, version 13, was used for this study. Following a review of the effects of single and combination impacts, the response surface method was employed to investigate the situation and evaluate the influence of treatment parameters. In this case, the independent variables (factors) were the coagulant dosage and mixing time. The three levels of these components are shown in Table 2: low level (−1), middle level (0), and high level (+1). The coded level values for these characteristics were selected based on a pilot study. Table 2 below also displays the matrix of the experimental design.

**Table 2:** Complete experimental design matrix of CCD.

Variables	Factor coding	UNITS	Level		
			−1	0	1
Coagulant dose	A	Mg/L	2	5	8
Mixing time	B	- min	20	40	60

## 3 Results and discussions

### 3.1 Raw textile wastewater effluent characterization

The investigation into the initial characteristics of the wastewater sample from the textile industry produced the following findings. As a result, the effluent's pH was determined to be 8.5 in the samples that were collected. This suggested that the textile industry's wastewater is more naturally alkaline. Total dissolved solids were measured at 4000 mg/L and total suspended solids at 1750 mg/L, respectively. Additionally, the investigation demonstrated that the textile wastewater sample contains more TDS particles than TSS particles. Additionally, the total solids in the wastewater sample that were experimentally determined were 5750 mg/L 235 NTU is the turbidity value at the end. In other words, textile wastewater includes three to four times as much organic content that can be broken down as non-organic matter. Preliminary characteristics of a textile wastewater sample were summarized in the following table.

Features of untreated textile wastewater are presented in Table 3. They show the necessity of developing treatment techniques. The textile industry discharges huge amounts of water, which gets heavily polluted with several impurities. One prominent feature of untreated textile wastewater is its extremely high turbidity, which amounts to about 235 NTU. This is very high, considering that wastewater should ideally not exceed 16 NTU. The turbidity indicates a very high concentration of suspended solids (TSS), which was found to be about 1750 mg/L.

**Table 3:** Characteristics of Kombolcha textile wastewater before treatment.

No	Parameters	Unit	Value
1.	TSS	mg/L	1750 ± 5
2.	TDS	mg/L	4000 ± 5
3.	TS	mg/L	6000 ± 5
4.	Turbidity	NTU	235 ± 5
5.	pH	–	8.5
6.	BOD,	mg/L	480 ± 5
7.	COD,	mg/L	1340 ± 5
8.	Conductivity	μS/cm	5000 ± 5
9.	Dyes (Residual Color):	Disperse, or azo dyes,	

### 3.2 Statistical analysis for *Moringa* coagulant on textile waste water treatment

The textile waste water is used for this analysis method. The removal of turbidity and TDS was calculated and measured accordingly. Design software was used to compile the measured data of the waste water sample for the coagulation studies into the following ANOVA table. Design expert 13.1.0.1 software was used to analyze the independent parameters of coagulant dose and mixing time. The elimination of TDS and turbidity from wastewater, as revealed by the percentage removal of turbidity (%), was the study's dependent variable.

#### 3.2.1 Analysis of variance (ANOVA) for percentage removal of TDS and Turbidity using *Moringa*

The following table shows the experimental result of *Moringa* olifera for textile wastewater.

The effects of mixing time and coagulant dosage on the performance of Moringa coagulant in treatment of textile wastewater were evaluated. The results show that the mixing time and dosage is quite significant in determining the efficacy of Moringa coagulant. Using a large mixing time and the highest dosage of coagulant, the turbidity level is decreased (not too bad, and the TDS level is also reduced in to 2000 mg/L. Additionally, obtained from the coagulation test was analyzed using design expert software as shown in Table 4. The coagulant dose (mg/L) and mixing time (min) are the two primary independent experimental procedure parameters in the test. TDS (mg/L) and the percentage of turbidity reduction (NTU) were the dependent variables employed as response parameters. From the results, the highest turbidity (NTU/l) and TDS (mg/L) removal efficiency was 65.3 % and 76.5 % respectively (at the dose of 2 mg/L, mixing time = 40 min.

**Table 4:** Experimental values of *Moringa* coagulant on textile waste water.

Std	Run	Factor 1	Factor 2	Response 1	Response 2
		A:coagulant dose	B:mixing time	turbidity	TDS
		mg/L	mini	NTU	mg/L
11	1	2	40	120	910
16	2	5	40	125	950
17	3	5	40	120	958
5	4	2	40	130	960
14	5	5	40	136	978
15	6	5	40	130	980
8	7	8	40	165	1050
10	8	5	60	163	1100
1	9	2	20	164	1158
2	10	8	20	165	2000
6	11	8	40	125	2050
13	12	5	40	185	2096
7	13	2	40	186	2159
9	14	5	20	152	1500
4	15	8	60	162	1522
12	16	5	60	172	1200
3	17	2	60	188	2100
20	18	3	40	128	2052
21	19	7	40	132	2117
18	20	5	60	165	1637
19	21	5	20	162	1620

**Fig. 1:**
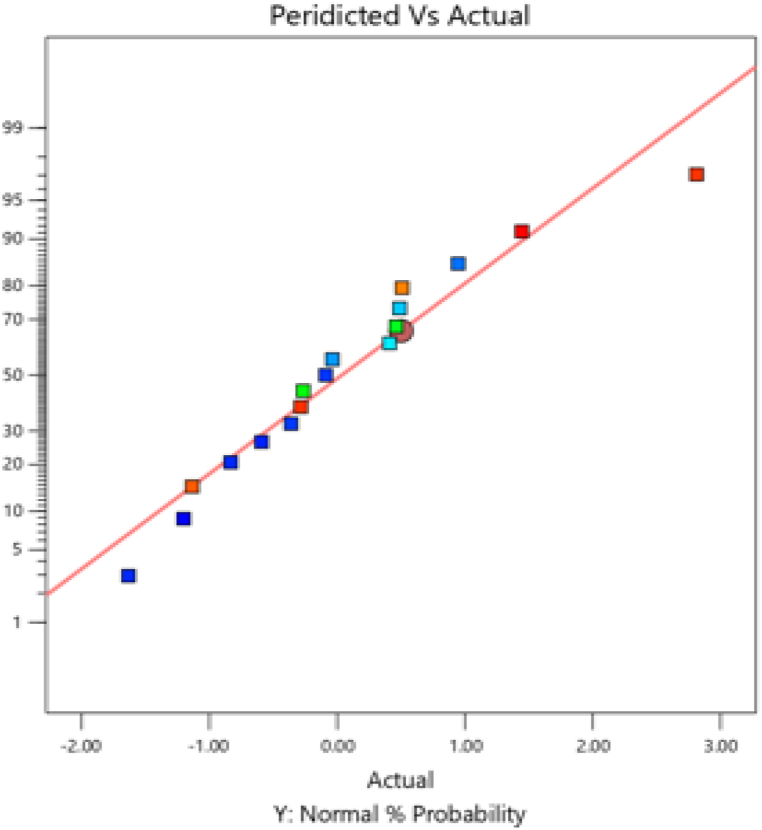
Predicted vs. Actual analysis of the turbidity removal process.

The turbidity removal process predicted vs. actual result is presented in Fig. 1 further down. The plot of projected against actual value is another crucial factor to consider when evaluating the model's suitability. If the scatter of the plot practically touches the diagonal line, this indicates that the model is well-designed since it means that the experimental data and the data predicted by the model are closely related and the experimental design analysis is expressed correctly. Therefore, "Predicted vs. Actual Analysis of the Turbidity Removal Process" compares the predicted turbidity removal values with actual experimental results. The data points align closely with a diagonal line, suggesting a strong correlation and model accuracy in predicting the turbidity removal performance.

#### 3.2.2 Statistical analysis for percentage removal of Turbidity (%)

According to the fit summary and as we observed in Table 5, a quadratic model that fits the chosen model the best and is appropriate for this investigation is one with a sequential p-value of less than 0.0001. The corrected R^2^ of 0.9873 and the expected R^2^ of 0.9455 are reasonably in agreement; that is, the difference is less than 0.2. The design space can be explored with this model. Table 5 presents the fit summary for the percentage removal of turbidity, highlighting the suitability of different models for this investigation. Of all the models evaluated, the quadratic one stands out as the most suitable. It gets a sequential p-value of less than 0.0001, which means it's significant. Because it is reliable and can predict outcomes effectively within the design space. This outcome is closely aligned with the findings reported by Ref. [20]. In plain contrast, the linear model and the two-factor interaction (2FI) models have p-values that stink. They have sequential p-values of 0.3810 and 0.5951, which mean they are not significant at all. Even though the cubic model is more complex, it is aliased and fails to provide meaningful insights, rendering a sequential p-value of 0.8433 and negative R^2^ values. Therefore, the quadratic model is recommended for exploring the design space associated with turbidity removal. This is due to the model's performance being better and the predictive accuracy being superior in contrast with the cubic model.

**Table 5:** Fit summary percentage removal of turbidity.

Source	Sequential *p*-value	Lack of Fit *P*-value	Adjusted R^2^	Predicted R^2^	
Linear	0.3810	0.7368	0.9873	−0.2604	
2FI	0.5951	0.6718	−0.0483	−0.6541	
Quadratic	**0.0001**	**0.0104**	**0.9873**	**0.9455**	**Suggested**
Cubic	0.8433	0.8318	−0.0844	−0.6179	**Aliased**

#### 3.2.3 Analysis of variance (ANOVA) for percentage removal of Turbidity (%)

Table 8's analysis of variance (ANOVA) revealed that, for varying values of independent variables, the total suspended solid removal values differed significantly (P ≤ 0.0500). In this instance, the model's F-value of 165.17 indicates that it is significant. Compared to the pure error, the lack of fit is insignificant, as indicated by the lack of fit F-value of 3.38. The probability of a substantial lack of fit F-value is increased by 10.40 % due to noise.

##### 3.2.3.1 Response 1: Turbidity

Predicted R^2^ and adjusted R^2^ values for a successful model should be close to each other and near 1.

Turbidity removal efficiency's ANOVA results are summarized in Table 6. The model's p-value (0.0428) indicates that, significantly, that is, it is a good representation of the data. The factors that are in the model (coagulant dose [A], mixing time [B], and their interaction [AB]) have a joint effect on turbidity removal efficiency. It have a very good model, and we can use it to predict how turbidity removal efficiency is going to react to changes in how much coagulant is used, the mixing time. Based on Table 6, the model is significant about the noise, according to the model's F-value of 0.046. Model terms are considered significant when the P-value is less than 0.0500. There are important model terms in this instance. The model terms are not significant if the values are higher than 0.1000. Model reduction could improve your model if it has many unnecessary terms (apart from those needed to maintain hierarchy). In comparison to the pure mistake, the Lack of Fit is not substantial, according to the Lack of Fit F-value of 0.13. A significant Lack of Fit F-value has a 93.79 % probability of being caused by noise. We want the model to fit, thus a non-significant lack of fit is ideal.

**Table 6:** ANOVA for percentage removal of turbidity (%).

Source	Sum of Squares	df	Mean Square	F-value	*p*-value	
Model	3741.30	5	748.26	165.46	0.0428	significant
A-coagulant dose	153.13	1	153.13	0.2546	0.0238	
B-mixing time	1176.13	1	1176.13	1.96	0.0095	
AB	6.25	1	6.25	0.0104	0.006	
A^2^	1616.35	1	1616.35	2.69	0.094	
B^2^	666.68	1	666.68	1.11	0.3149	
Residual	6614.58	11	601.33			
Lack of Fit	313.38	3	104.46	3.3826	0.1040	not significant
Pure Error	6301.20	8	787.65			
Core Total	10355.88	16				

#### 3.2.4 Statistical analysis for percentage removal of TDS

Because the suggested quadratic model for TDS (mg/L) has a sequential p-value of less than 0.0001 as shown in Table 7, and a higher predicted and adjusted R^2^ value, the selected quadratic model can be used for this study. This suggested model has adjusted and predicted R^2^ values of 0.9900 and 0.9340, respectively, as shown in Table 7. The model is important, which implies it can accurately reflect this task, according to the criteria mentioned above. Table 7 précises the model fit for the percentage removal of TDS, highlighting the quadratic model as the most suitable. The difference between adjusted and predicted R^2^ is less than one-fifth, reinforcing our confidence in the model's performance. This stands in sharp contrast to the performance of the linear and 2FI models, which have sequential p-values of around 0.1 and negative adjusted R^2^ values that reflect significantly low accuracy. The cubic model, on the other hand, is aliased and, thus, not suitable for further analysis.

**Table 7:** Fit summary percentage removal of TDS.

Source	Sequential *p*-value	Lack of Fit *P*-value	Adjusted R^2^	Predicted R^2^	
Linear	0.9587	0.6094	−0.1360	−0.5118	
2FI	0.1839	0.7024	−0.0624	−0.5476	
Quadratic	**0.0001**	**0.2211**	**0.9900**	**0.9340**	**Suggested**
Cubic	0.9109	0.7796	−0.1276	−0.9192	Aliased

#### 3.2.5 Analysis of variance for percentage removal of TDS (%)

The F-value of 209.92 at p < 0.0001 illustrates the quadratic model's considerable character. If the p-value is less than 0.05, the model terms are likewise statistically significant. Additionally, compared to the pure error, the lack of fit's F-value of 2.07 indicates that it is not significant. The likelihood that noise is the cause of a lack of fit F-value is 28.11 %. As a result, this model has no fit issues.

The statistical analysis of the wastewater treatment model was performed, and the results are summarized in Table 8. The model is significant (p < 0.0001, F = 209.92), unlike the individual factors it evaluates (A and B), which are not significant at all (p = 0.8590 and 0.7976, respectively). The model carves out a fairly good fit, or explanation, of how linear (A and B) and interaction (AB) terms, along with some quadratics (A^2^, B^2^), influence treatment performance. But a closer look at residuals (p = 0.281) suggests the factors, while they influence performance, don't seem to have independent (or significant) effects on performance.

**Table 8:** ANOVA for percentage removal of TDS (%).

Source	Sum of Squares	df	Mean Square	F-value	*p*-value	
Model	1.358E+06	5	2.717E+05	209.92	<0.0001	significant
A-coagulant dose	7503.12	1	7503.12	0.0331	0.8590	
B-mixing time	15664.50	1	15664.50	0.0690	0.7976	
AB	5.041E+05	1	5.041E+05	2.22	0.1642	
A^2^	8.020E+05	1	8.020E+05	3.54	0.0868	
B^2^	14495.04	1	14495.04	0.0639	0.8051	
Residual	2.496E+06	11	2.269E+05			
Lack of Fit	76534.07	3	25511.36	0.0844	0.281	not significant
Pure Error	2.419E+06	8	3.024E+05			

**Fig. 2:**
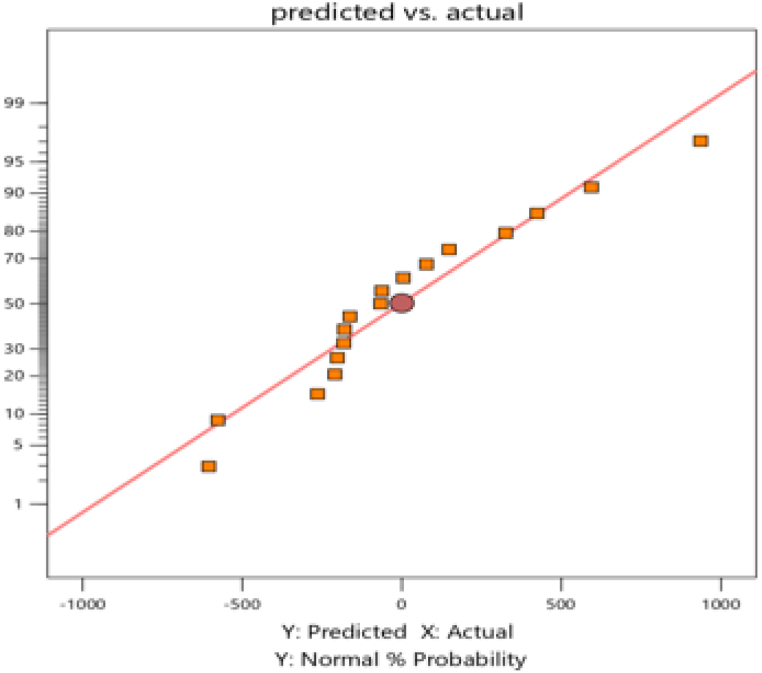
Predicted vs. actual plot.

The total dissolved solid removal process predicted vs. actual result is shown in Fig. 2 below.

The plot of projected against actual value is another crucial factor to consider when evaluating the model's suitability. Suppose the scatter of the plot practically touches the diagonal line. In that case, this indicates that the model is well-designed since it means that the experimental data and the data predicted by the model are closely related as we saw in Fig. 2.

#### 3.2.6 Effect of independent variables on percentage removal of total dissolved solids (TDS)

By adding them in several amounts and levels to the *Moringa* coagulant dose, the impact of coagulant dosage (mg/L) and mixing time (min) on the removal efficiency of total dissolved solids (TDS) was examined.

##### 3.2.6.1 Effect of coagulant dosage (mg/L) on percentage removal of TDS

By varying the dose (mg/L) of prepared cactus pad coagulant the removal efficiency of total dissolved solids (TDS) was examined. The figure illustrates the impact of coagulant dosage on total dissolved solids (TDS) removal efficiency. It demonstrates how the coagulant influences TDS reduction efficiency.

**Fig. 3:**
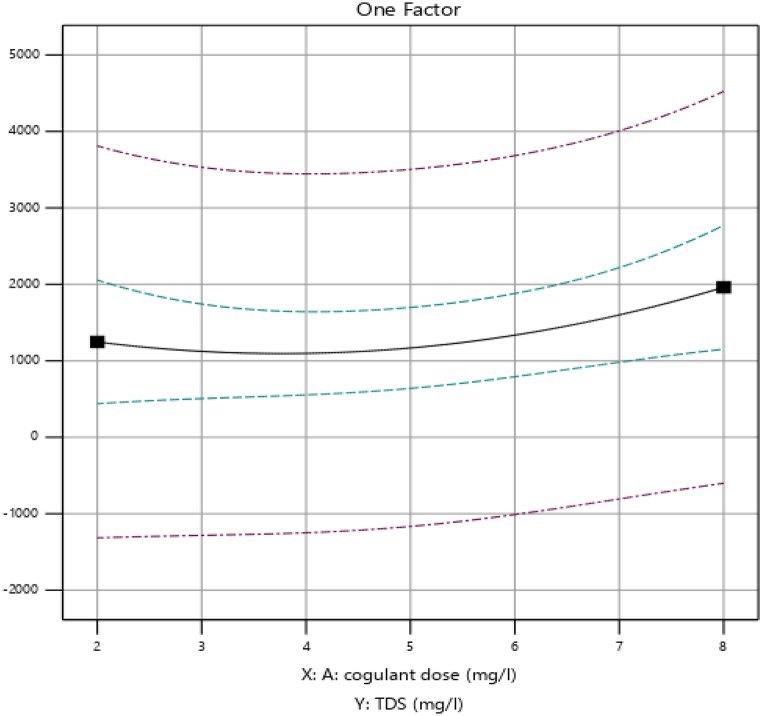
Effect of coagulant dosage (mg/L) on percentage removal of TDS (%).

As we observed in Fig. 3, the dose increased from 2 mg/L to 5 mg/L, the percentage of TDS (mg/L) removed from the wastewater sample is almost constant and then 5 mg/L to 8 mg/L, it is increased from 72.2 % to 76.5 %. This increase is the result of the coagulant's active site preference increasing.

##### 3.2.6.2 Effect of mixing time (min) on percentage removal of TDS

**Fig. 4:**
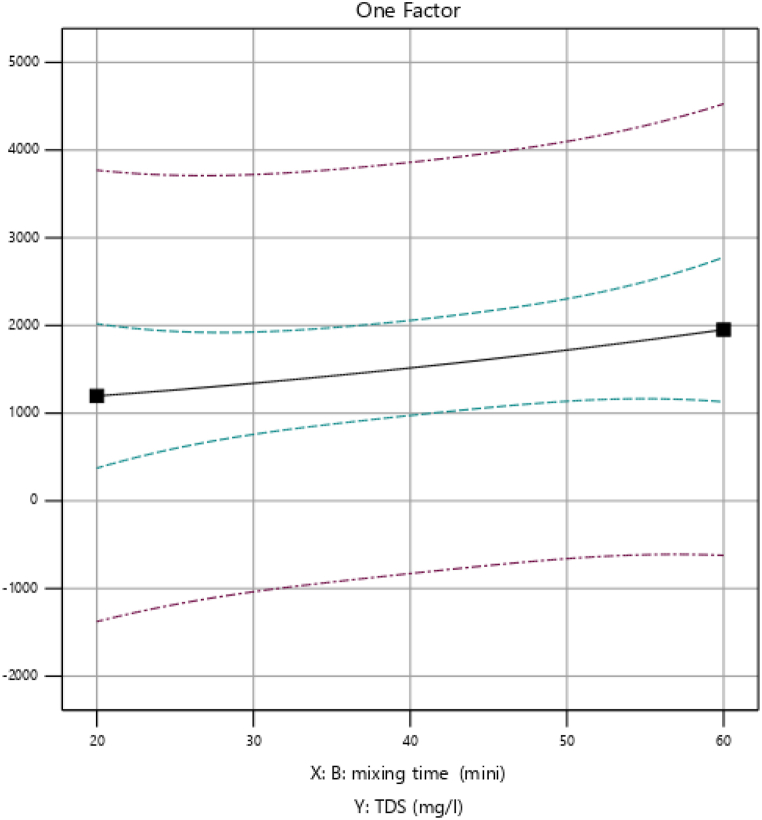
Effect of mixing time (min) on percentage removal of TDS.

As presented in Fig. 4, the removal efficiency of TDS (mg/L) increased from 74.9 % to 76.5 %, as the mixing time increased from 20 to 60 min. This is because increasing the mixing time will enhance the rate of collision and coagulation of particles. However, increasing mixing time beyond 20 min reduces TDS (mg/L) removal from 76 % to 75.6 %. Therefore as the mixing time increases the removal efficiency increases as we observed in the figure below.

The figure provided shows that the removal efficiency of Total Suspended Solids (TDS) increases significantly with longer mixing times. This increase is primarily due to the longer coagulant/colloidal particle interaction times afforded by the longer mixing durations, leading to better results. Mixing times that enhance TDS removal are, therefore, critical in TDS-removal water treatment processes. This result closely corresponds to the findings reported by Ref. [12].

#### 3.2.7 Effect of independent variables on percentage removal of Turbidity

Applying coagulant dosage (mg/L) and mixing time (min) in varying amounts and levels on the dose of *Moringa* coagulant allowed for the investigation of their effects on the removal effectiveness of turbidity.

##### 3.2.7.1 Effect of coagulant dosage (mg/L) and mixing time on percentage removal of Turbidity

**Fig. 5:**
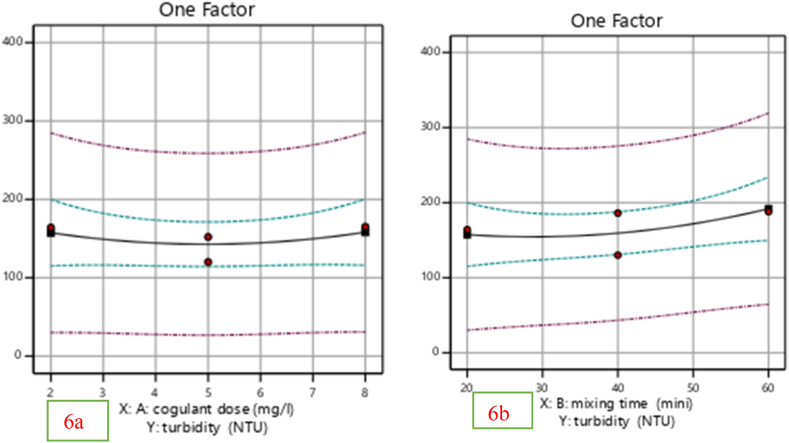
Effect of coagulant dose (a) and mixing time (b) on percentage removal of turbidity.

The effectiveness of the produced *Moringa* coagulant and mixing time in removing turbidity was tested by changing the dose (mg/L). Fig. 5a under shows that the percentage of Turbidity eliminated from the wastewater sample slightly increased when the dose slightly increased at 2 mg/L and then almost constant from 5 mg/L to 8 mg/L. The coagulant's predilection for the active site is growing, which is the cause of this increase. And also Mixing time (min) has a considerable effect on the removal efficiency of turbidity and other pollutants in wastewater treatment.

From Fig. 5b. The turbidity gets improved from 47.9 % to 52.9 % as the mixing time (min) continued to rise from 40 min to 50 min. According to the study done by Ref. [21] suitable mixing time (min) must be provided to allow the production of particles of large size to permit their efficient removal in wastewater. This reduction of removal efficiency may be due to the re-stabilization of particles in the wastewater.

The key factors that affect turbidity removal efficiency are illustrated in Fig. 5a and b. Coagulant dosage is depicted in Fig. 5a, where it can be seen that turbidity removal efficiency improves with increased coagulant dosage up to a certain threshold. If the coagulant dose is inadequate, the inconsistently charged particles in the suspension do not agglomerate; if too much coagulant is used, mixing time must be sufficient to ensure that the settling of agglomerates does not occur in a portion of the reactor where turbulent flows may still be present. Agitator speed, seen in Fig. 5b, is an important factor that influences mixing time and this discussion is closely related to the findings presented by Ref. [22].

#### 3.2.8 Interaction effects of independent variables on Textile wastewater treatment

Using design expert software, the potential effects of independent experimental variables on the removal efficiency of TDS (mg/L) and turbidity (mg/L) were illustrated. Varied interactions between the variables are indicated by a varied 3D surface plot shape. By holding the third variable constant, these charts show the relative impact of any two variables.

##### 3.2.8.1 Interaction effects on percentage removal of TDS and Turbidity

###### 3.2.8.1.1 Interaction effect of dose of coagulant (mg/L) and mixing time (min) on TDS

**Fig. 6:**
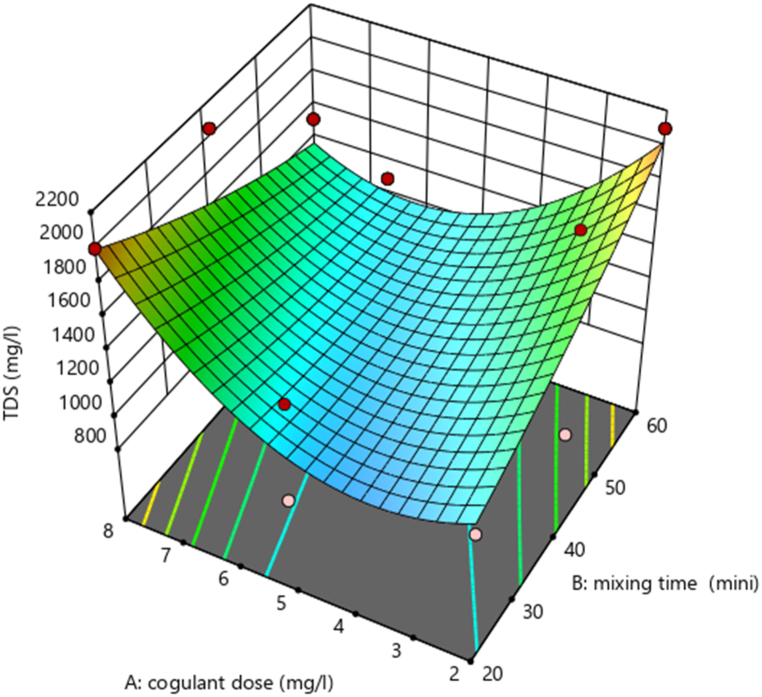
Interaction effect of mixing time (min) and dose (mg/L) on removal of TSS (%).

The elimination efficiency of TSS (mg/L) is marginally impacted by the interaction between AB (mixing time (min) and coagulant dose (mg/L), according to Table 6 ANOVA analysis. To investigate the AB (mixing time (min)) interaction effect. As the dose (mg/L) and mixing time (min) increased up to 8 mg/L and 60 min, respectively, the removal efficiency of TSS (mg/L) increased, as shown in Fig. 6.

**Fig. 7:**
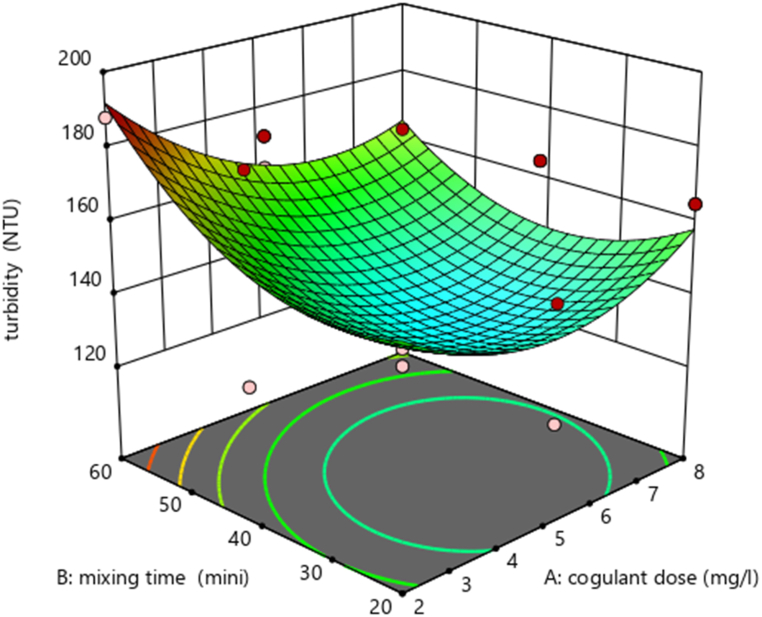
Interaction effect of mixing time (min) and dose (mg/L) on the removal of Turbidity (%).

**Fig. 8:**
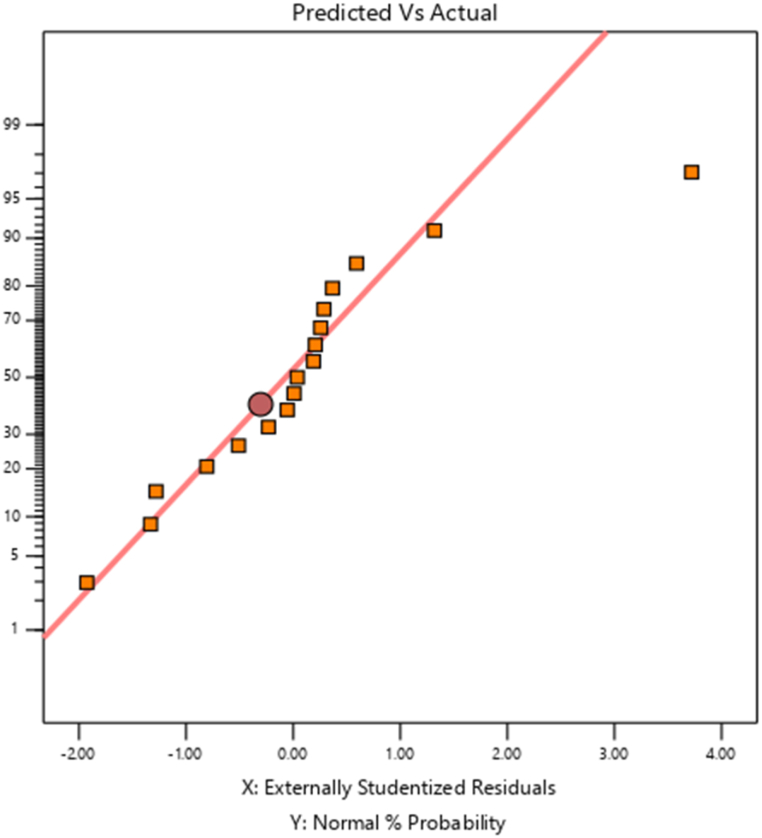
Predicted vs. actual plot of the model.

The interaction effect between mixing time (in minutes) and dose (in mg/L) on the removal efficiency of Total Suspended Solids (TSS) is depicted in the figure (Fig. 6) (see Fig. 8). This effect suggests that these two parameters have a powerful influence on the removal percentages of the TSS. It is almost as if they were two members of a band, playing different but complementary parts, and together reaching an optimal performance that is almost as good as it gets for TSS removal. As you can see, moving from left to right along the x-axis, which represents the mixing time in minutes, and to a certain point across the y-axis, which represents the dose in mg/L (from the bottom up), you achieve what might be called maximum TSS removal efficiency.

###### 3.2.8.1.2 Interaction effect of dose of coagulant (mg/L) and mixing time (min) on Turbidity

The graph shows the interaction effect between coagulant dose and mixing time on the removal efficiency of turbidity and also the read peak point in the graph shows the removal efficiency is high. Interaction between AB (mixing time (min) and coagulant dose (mg/L)) has a minimal impact on Turbidity removal efficiency (mg/L), according to Table 6 ANOVA analysis. To investigate the AB (mixing time (min)) interaction effect. As the value of the dose (in mg/L) and mixing time (in min) increased up to 8 mg/L and 60 min, respectively, the removal efficiency of Turbidity (in mg/L) is high. (Fig. 7).

### 3.3 Statistical analysis for Cactus pads coagulant on textile wastewater treatment

As discussed in section 3.2’ this analysis method makes use of textile wastewater. Turbidity and TDS reduction were computed and measured appropriately. The design software was used to compile the measured values of the wastewater sample for the coagulation/flocculation studies into the following ANOVA table. The independent parameters that were analyzed with Design Expert 13.1.0.1 software were the dosage of the coagulant and the duration of mixing. The removal of TDS and turbidity from wastewater, as determined by the percentage of turbidity removed (%), was the dependent variable in this study. The following table shows the experimental result of cactus pads for textile wastewater with star points.

The experimental results featured in Table 9 center on the cactus pad coagulant's effectiveness in treating textile wastewater. With this study, we aimed to determine the optimal coagulant dosage and mixing time to achieve the best possible results regarding Total Dissolved Solids (TDS) and turbidity reduction, two common problems seen in not just textile wastewater but also many other types of wastewater. The results indicate that both these parameters significantly affect the TDS and turbidity results.

**Table 9:** Experimental values of cactus pad coagulant on textile wastewater.

Std	Run	Factor 1	Factor 2	Response 1	Response 2
		A:coagulant dosage	B:mixing time	TDS	Turbidity
		mg/L	min	mg/L	mg/L
13	1	2	40	810	120
1	2	2	40	600	110
7	3	2	40	820	120
14	4	5	40	860	130
4	5	5	60	978	200
3	6	5	60	980	190
10	7	5	60	1050	185
11	8	5	20	660	163
12	9	5	60	1158	190
16	10	5	40	850	165
8	11	8	40	880	125
17	12	5	40	810	185
9	13	5	20	690	186
6	14	8	40	900	152
5	15	8	40	950	162
15	16	8	40	810	172
2	17	8	20	720	188
18	18	1	40	765	180-
19	19	9	40	736	185
20	20	5	12	747	183
21	21	5	68	741	184

#### 3.3.1 Statistical analysis for percentage removal of TDS

The selected liner model can be employed for this investigation since the proposed model for TDS (mg/L) has a higher predicted and adjusted R^2^ value and a sequential p-value of less than 0.0001. The table displays the adjusted and predicted R^2^ values for this proposed model, which are 0.9700 and 0.9040, respectively. The significance of the model suggests that it can faithfully capture this task based on the previously stated criteria.

#### 3.3.2 Analysis of variance for percentage removal of TDS (%)

The high importance of the liner model is demonstrated by the F-value of 58.47 at p < 0.0001 as we observed in Table 11. Additionally, the model terms are statistically significant if the p-value is less than 0.05. Furthermore, when compared to the pure error, the lack of fit's F-value of 2.07 shows that it is not statistically significant. The likelihood that an unfit F-value is due to noise is 97.17 %. Consequently, there is no fit problem with this model.

The significance of the model is indicated by its F-value of 58.47. An F-value of this magnitude is just 0.01 % likely to be the result of noise; model terms are relevant when the value is less than 0.0500. A and B are important model terms in this instance. As shown in Fig. 7, the model terms are insignificant if the values are higher than 0.1000. Model reduction could make your model better if it has a lot of unnecessary terms (apart from those needed to maintain hierarchy). In comparison to the pure mistake, the Lack of Fit is not substantial, according to the Lack of Fit F-value of 0.12. A significant Lack of Fit F-value has a 97.17 % probability of being caused by noise. We want the model to fit, thus a non-significant lack of fit is ideal.

#### 3.3.3 Effects of independent variables on Turbidity and TDS removal

When a cactus pad was used as a coagulant in textile wastewater treatment, the effects of the independent variables (particle size (mm) and coagulant dose (mg)) on the removal of total dissolved solids and turbidity were investigated.

##### 3.3.3.1 Effect of coagulant dosage (mg/L) on percentage removal of TDS

**Fig. 9:**
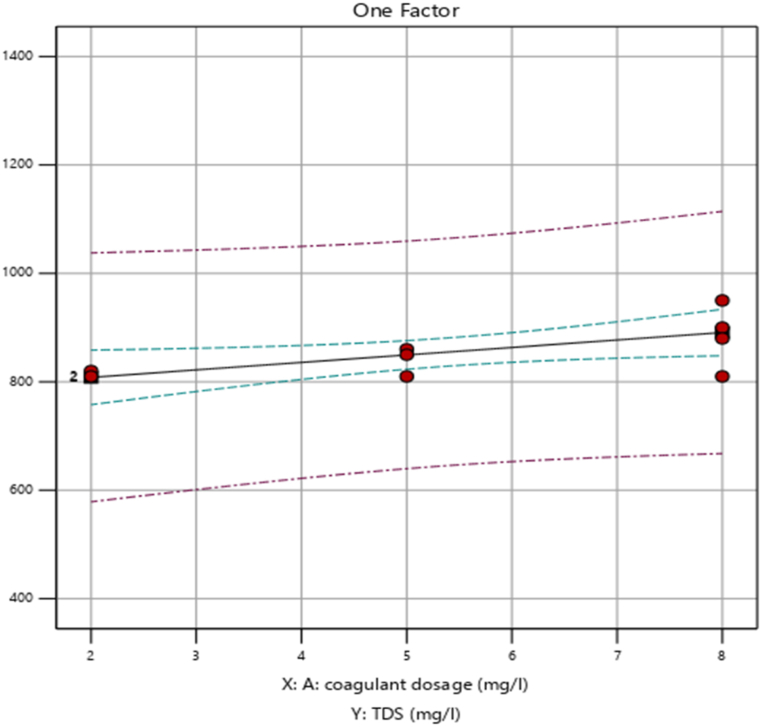
Effect of coagulant dosage (mg/L) on percentage removal of TDS (%).

By varying the dosage (mg/L), the efficiency of the generated cactus pad coagulant in removing total dissolved solids (TDS) was evaluated. When the dose was increased from 5 mg/L to 8 mg/L, the percentage of TDS (mg/L) removed from the wastewater sample rose from 72.2 % to 76.5 %, as presented in Fig. 9. This rise is due to the coagulant's increasing preference for the active site. This rise is due to the coagulant's increasing preference for the active site. However, when the coagulant dose was increased over 5 mg/L, the removal efficiency of TDS (mg/L) decreased to 50.8 %. This conclusion is supported by the research conducted by Ref. [23]. Because low coagulant dosages, which also have a weak coagulation impact, lead to inadequate coagulation. An excessive amount of coagulant encloses the particles in the raw water, causing their surfaces to become saturated. This reduces the number of particles that can mix during coagulation and achieve stability.

##### 3.3.3.2 Effect of mixing time (min) on percentage removal of TDS

**Fig. 10:**
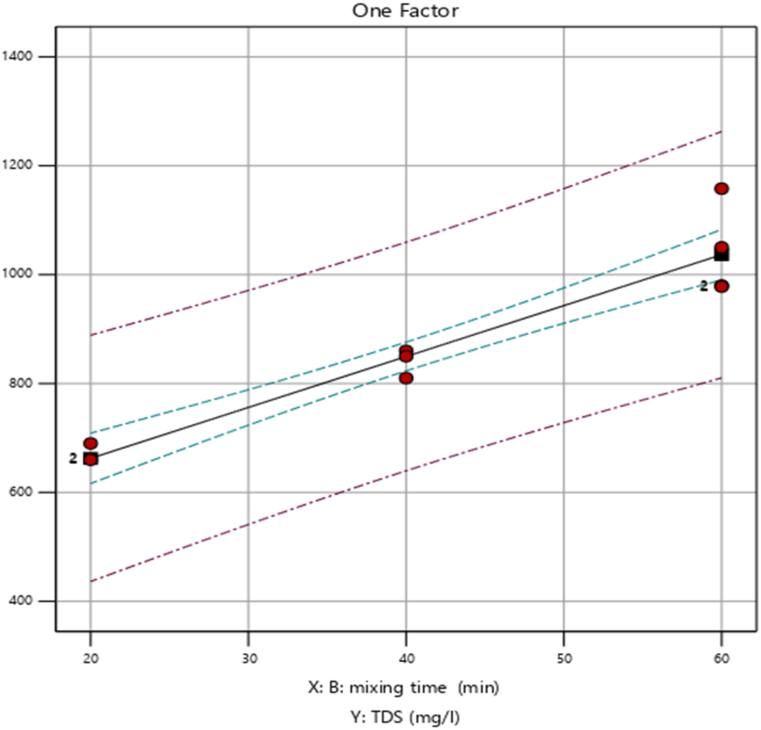
Effect of mixing time (min) on percentage removal of TSS.

As the mixing duration extended from 20 to 60 min, the elimination efficiency of TSS (mg/L) increased from 74.9 % to 76.5 %, as illustrated in Fig. 10. This is so that the rate of particle collision and coagulation can be increased by prolonging the mixing time. However, mixing times of less than 20 min resulted in a reduction of TDS (mg/L) removal from 76 % to 75.6 %. This suggests once more that TDS (mg/L) removal effectiveness starts to decrease as mixing time or length increases above 60 min [24]. found that aggregates will be exposed to fluid shear rates for an extended length of time with greater mixing durations. Delaying the floc development process and extending the time for particle contact, will raise the rate of floc breakup.

The noted increase in TDS removal with longer mixing times this discussion is a good agreement with the previously reported result [25] in which extended mixing is shown to enhance the mass transfer and the dissolution of coagulants in solution. If mixing times are too short, soluble salts break down too slowly and the treatment system doesn't achieve nearly as high an overall TDS reduction efficiency.

#### 3.3.4 Effect of independent variables on percentage removal of Turbidity

Applying coagulant dosage (mg/L) and mixing time (min) in different amounts and levels on the dose of cactus pad coagulant allowed for the assessment of their effects on the removal efficacy of turbidity.

##### 3.3.4.1 Effect of coagulant dosage (mg/L) and mixing time on percentage removal of Turbidity

**Fig. 11:**
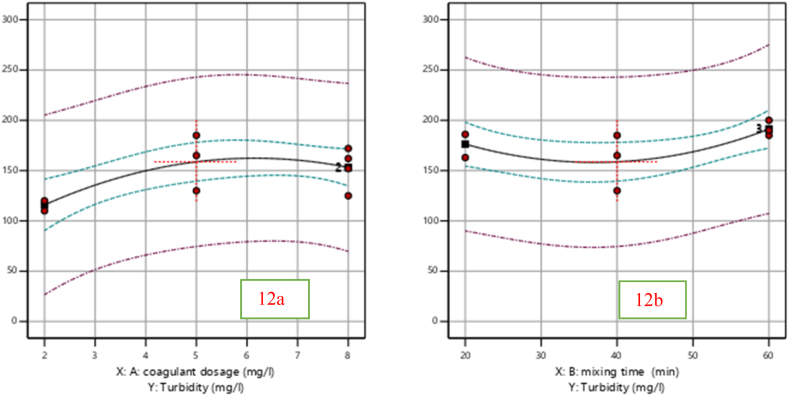
Effect of coagulant dosage (mg/L) (a) and mixing time (b) on percentage removal of TDS (%).

By varying the dose (mg/L), the efficacy of the generated cactus pad coagulant and mixing time in eliminating turbidity was evaluated. Fig. 11a illustrates how the dose increased from 5 mg/L to 8 mg/L, slightly increasing the proportion of turbidity removed from the wastewater sample. This rise is due to the coagulant's increased preference for the active site. Additionally, the effectiveness with which turbidity and other pollutants are removed during wastewater treatment is significantly impacted by the mixing time (min). Fig. 11b illustrations that when the mixing time (min) increased from 40 to 50 min, the turbidity improved from 47.9 % to 52.9 %. According to the study done by Ref. [21] suitable mixing time (min) must be provided to allow the production of particles of large size to permit their efficient removal in wastewater. As [26] explained higher circulation times reduce the time aggregates have to form larger structures before fragmentation induced by the high shear stress.

#### 3.3.5 Interaction effects on percentage removal of TDS and Turbidity

##### 3.3.5.1 Interaction impact of dose of coagulant (mg/L) and mixing time (min) on TDS

**Fig. 12:**
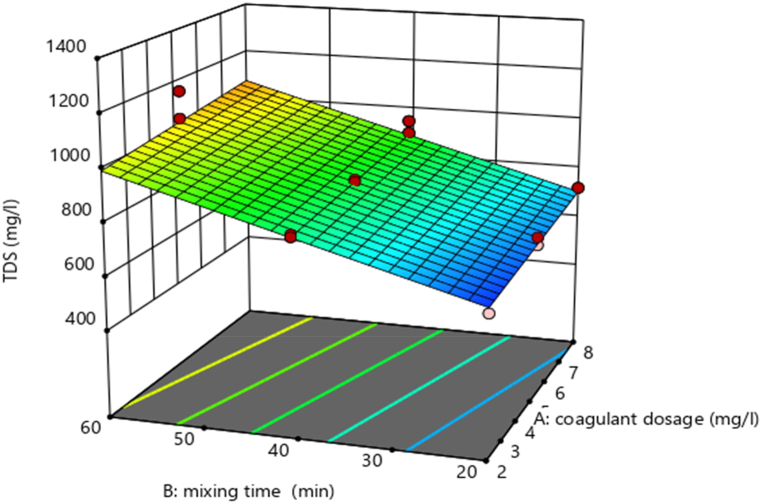
Interaction effect of mixing time (min) and dose(mg/L) on removal of TSS (%).

The interaction between AB (mixing time (min) and coagulant dose (mg/L)) has a minimal impact on TSS removal efficiency (mg/L), according to Table 11, ANOVA analysis. To investigate the AB (mixing time (min)) interaction effect. As we observed in Fig. 12. The value of the dose (in mg/L) and mixing time (in min) increased up to 8 mg/L and 60 min, respectively, the removal efficiency of TSS (in mg/L) increase than the other dose and mixing time.

##### 3.3.5.2 Interaction effect of dose of coagulant (mg/L) and mixing time (min) on Turbidity

**Fig. 13:**
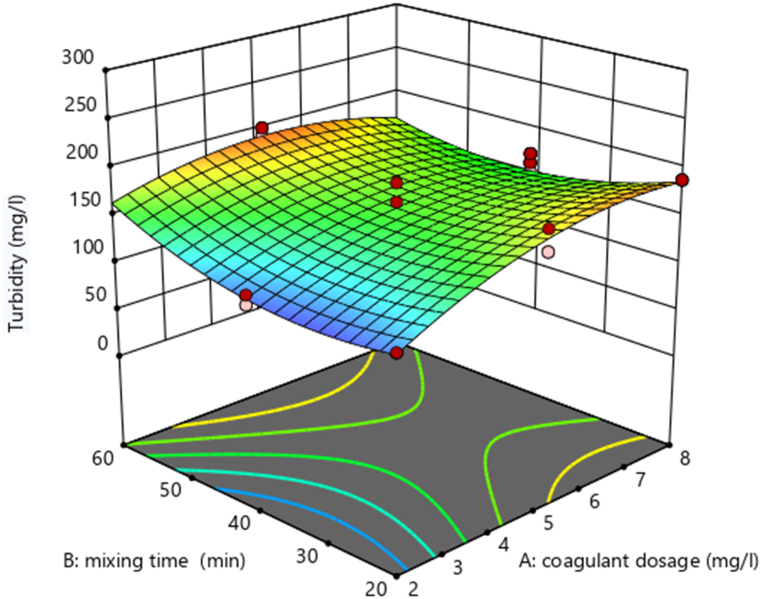
Interaction effect of mixing time (min) and dose (mg/L) on the removal of Turbidity (%).

The interaction effect of mixing time (in minutes) and dose (in mg/L) on turbidity removal efficiency (%) is also illustrated by the figure Fig. 13 below. The curve appears to show an optimal interaction of the two variables, where maximum turbidity removal is occurring. When you plot turbidity removal against the two parameters (mixing time and dose. Table 8 ANOVA analysis shows that the interaction between AB (mixing time (min) and coagulant dose (mg/L)) does not affect TSS removal efficiency (mg/L). To look at the interaction effect of AB (mixing time (min)). Turbidity removal efficiency (measured in mg/L) rose when the dose (measured in mg/L) and mixing time (measured in min) increased to 8 mg/L and 60 min, respectively (Fig. 13).

### 3.4 Comparative analysis of *Moringa* seed and Cactus pads efficiency on textile wastewater treatments

#### 3.4.1 The optimum operational parameters

As the coagulant's active site preference increased, the ideal conditions for removing turbidity and TDS utilizing *Moringa* seed as a natural coagulant in the coagulation process were determined using Anova Table 10, Table 11 analysis. However, when the coagulant dose was increased above 5 mg/L, the efficacy of TDS (mg/L) elimination decreased to 75.8 %. However, TDS (mg/L) clearance decreases from 76 % to 75.6 % when mixing duration and coagulant dose are increased beyond 60 min (see Fig. 11a). Demonstrates that when the dosage was raised from 5 mg/L to 8 mg/L, the percentage of turbidity removed from the wastewater sample slightly increased. The coagulant's predilection for the active site is growing, which is the cause of this increase. In addition, mixing time (min) has a considerable effect on the removal efficiency of turbidity and other pollutants in wastewater treatment. From Fig. 11b. The turbidity improved from 47.9 % to 52.9 % as the mixing time (min) continued to rise from 40 min to 50 min. Similarly, by varying the dosage (mg/L), the efficiency of the generated cactus pad coagulant's removal of total dissolved solids (TDS) was evaluated. When the dose was increased from 5 mg/L to 8 mg/L, the percentage of TDS (mg/L) removed from the wastewater sample rose from 72.2 % to 76.5 %, as displayed in Fig. 13. Therefore, to get the best performance out of both natural coagulants, these ideal experimental circumstances are required.

**Table 10:** Fit summary percentage removal of TDS.

Source	Sequential *p*-value	Lack of Fit *p*-value	Adjusted R^2^	Predicted R^2^	
Linear	**< 0.0001**	**0.9717**	**0.9700**	**0.90421**	**Suggested**
2FI	0.5605	0.9756	0.8719	0.8433	
Quadratic	0.9567	0.7333	0.8499	0.8019	
Cubic	0.7333		0.8368		**Aliased**

**Table 11:** ANOVA for percentage removal of TDS (%).

Source	Sum of Squares	df	Mean Square	F-value	*p*-value	
Model	2.930E+05	2	1.465E+05	58.47	< 0.0001	significant
A-coagulant dosage	13275.30	1	13275.30	5.30	0.0372	
B-mixing time	2.798E+05	1	2.798E+05	111.64	<0.0001	
Residual	35080.94	14	2505.78			
Lack of Fit	1621.94	4	405.48	0.1212	0.9717	not significant
Pure Error	33459.00	10	3345.90			
Core Total	3.281E+05	16				

Table 12 illustrates the above-stated premise most efficiently. It demonstrates that applying a coagulant at a concentration of 2 mg/L for 40 min a condition that was found to be optimal for both the coagulants under study shows a remarkable reduction in two of the most common effluent problems both turbidity and TDS. Gathering results helps to tell a more compelling and coherent story. With the cactus pad coagulant, at 2 mg/L for 40 min, TDS was reduced from a startling 4000 mg/L down to just 600 mg/L. As for turbidity, with the cactus pad coagulant at the same dosage and time interval, the study went from 235 NTU down to just 110 NTU.

As showing in Table 12, we ran 17 trials by change how much coagulant was used and how long things were stirred to figure out what worked best for clearing up cloudiness and getting rid of dissolved stuff. So, in those scenarios, we checked out, that using a little bit of coagulant at 2 mg/L with stirring for 40 min did the trick most effectively compared to other amounts and times. In addition to this, the best conditions for eliminating turbidity and TDS were 2 g/l and 40 min for the coagulant dose and mixing time, respectively, while employing *Moringa* seed and cactus pads as a natural coagulant in the coagulation process. Therefore, to get the best performance out of both natural coagulants, these ideal experimental circumstances are required. Overall, the data implies that both types of coagulants function well at 2 mg/L and 40 min, highlighting their possible usefulness in real-world scenarios of treating textile wastewater. This also gives us the green light to look deeper into plant-based coagulants as a viable alternative to petroleum-based coagulants that are not so eco-friendly.

**Table 12:**
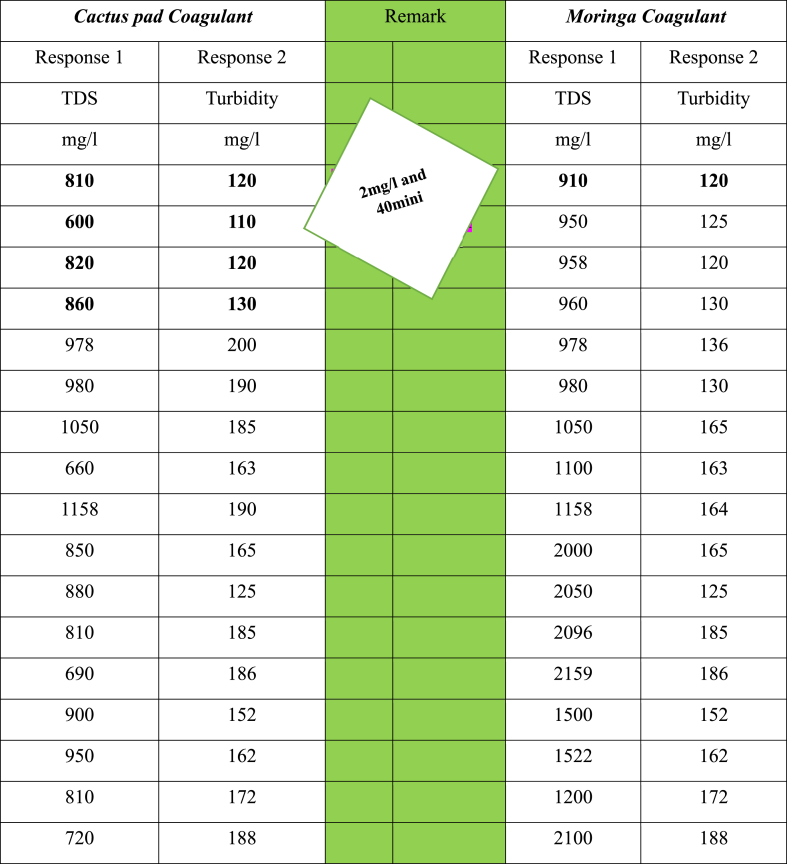
Comparisons between the cactus pad and *Moringa* coagulant on the efficiency of textile wastewater treatment.

Table 13 condenses a lot of information and presents it in a way that is easier to understand, summarizing the results of Coagulation (using both synthetic and natural coagulants) applied to real scenarios of treating textile wastewater. Full references cited in the table can be checked for specific values that lend more detail and context to the results.

**Table 13:** Value of parameters before and after treatment of coagulant.

Parameter	Before treatments	After Treatment with Synthetic Coagulants			After Treatment with natural Coagulants		
		Result	%Removal (Typical)	Reference	After treatments using *Moringa* seed	After treatments using cactus seed	(%) Removal at optimum values
Turbidity (NTU)	235 ± 5	10–50	50 %	[27]	160 ± 5	110 ± 5	53.19
TDS (mg/L)	4000 ± 5	500–1000	77.5 %	[28]	910 ± 5	120 ± 5	97.00
BOD (mg/L)	480 ± 5	100–250	47.5 %	[29]	201 ± 5	198 ± 5	58.75
COD (mg/L)	1340 ± 5	300–700	26.5 %	[30]	1005 ± 5	1000 ± 5	25.37
Conductivity (μS/cm)	5000 ± 5	1500–3000	57.5 %	[31]	2012 ± 5	2000 ± 5	60

Additionally, Table 13. Illustrate wastewater quality indicators that were compared before and after treatment with Moringa and cactus seeds as natural coagulants. The starting turbidity was 235 NTU. After treatment with Moringa seeds, the turbidity was reduced to 160 NTU. With the use of cactus seeds, the turbidity was reduced to 110 NTU, which is a 53.19 % removal efficiency. Neither treatment reached the Ethiopian discharge standard of ≤ 30 NTU, but the discharge from the cactus coagulant was closer. The next indicator was total dissolved solids (TDS); the starting TDS concentration was 4000 mg/L. Moringa achieved a reading of 910 mg/L after treatment, and the cactus coagulant achieved a reading of 120 mg/L. The TDS reading after treatment with cactus seeds had a removal efficiency of 97 %. While the cactus was the seed that was closer to meeting any standard, both types of treatment significantly improved the wastewater quality.

### 3.5 Limitations

Focusing on one industry and particular forms of wastewater could make our results less broadly applicable. Our undertaking of this research actually raises another issue: the impact of natural coagulants on seasonal variability. Simple quantities of natural coagulants could have significant effects during different times of the year. Finally, our work signals the need for a larger cost-benefit evaluation of natural coagulants if they are to be used on a scale that would truly impact the appearance of clean water in rivers and streams.

## 4 Conclusions

The necessity of treating textile wastewater was thrown into sharp relief by this investigation. Coagulation, the method used in the study, was demonstrably effective at removing nasty stuff from wastewater. It had an initial turbidity of 235 NTU (nephelometric turbidity units), a pH of 8.5, and a total solid content (TS) of 6000 mg/L. Of that, 3500 mg/L was dissolved solids (TDS), and 1750 mg/L was suspended solids (TSS). Several tasks were carried out to fulfill the aim of the study. The natural coagulants powdered *Moringa* seeds and cactus pads were tested and found to be extremely effective. They reduced the turbidity, total dissolved solids, and total suspended solids to the point where these could be classified as clean and safe to discharge. The data shows that cactus seed treatment is more effective than Moringa seed treatment for purifying water across all the measured criteria. Following treatment with the seeds of the cactus, the water had a turbidity of 110 NTU, total dissolved solids (TDS) of 120 mg/L, biochemical oxygen demand (BOD) of 198 mg/L, chemical oxygen demand (COD) of 1000 mg/L, and conductivity of 2000 μS/cm. In contrast, after treatment with *Moringa* seeds, the same water had turbidity of 160 NTU, TDS of 910 mg/L, BOD of 201 mg/L, COD of 1005 mg/L, and conductivity of 2012 μS/cm. Taken together, these variables indicate that the cactus seeds are a superior coagulant.

Natural coagulants certainly provide an excellent treatment of industrial wastewater, yielding a very high treatment efficiency. This study shows that they are also quite suitable for developing countries: they are inexpensive most are free, in fact so direct costs are low; and they are sustainable because they are made from renewable resources. Using natural coagulants thus addresses the challenge of environmental sustainability in two ways: first, because they themselves are environmentally friendly; and second, because their low cost greatly lessens the chance that wastewater treatment will be abandoned, which could happen if direct treatment costs are too high.

Future research ought to focus on combining cactus and *Moringa* with other treatment methods, like filtration and adsorption, to see if these methods improve treatment efficiency. Likewise, it would be beneficial to examine the performance of these two coagulants in various industrial situations.

## CRediT authorship contribution statement

**Getahun Demeke Worku:** Writing – review & editing, Writing – original draft, Methodology. **Shimeles Nigussie Abate:** Writing – review & editing, Validation.

## Data availability statement

Data included in the article/supplementary material is referenced in the article. The data supporting this study's findings are available from the corresponding author upon reasonable request.

## Funding

This research did not receive any specific grant from funding agencies in the public, commercial, or not-for-profit sectors.

## Declaration of competing interest

The authors declare that they have no known competing financial interests or personal relationships that could have appeared to influence the work reported in this paper.
